# Genome sequence of the squalene-degrading bacterium *Corynebacterium terpenotabidum* type strain Y-11^T^ (= DSM 44721^T^)

**DOI:** 10.4056/sigs.4588337

**Published:** 2013-12-31

**Authors:** Christian Rückert, Andreas Albersmeier, Arwa Al-Dilaimi, Hanna Bednarz, Karsten Niehaus, Rafael Szczepanowski, Jörn Kalinowski

**Affiliations:** 1 Technology Platform Genomics, CeBiTec, Bielefeld University, Bielefeld, Germany; 2 Proteome and Metabolome Research, Bielefeld University, Bielefeld, Germany

**Keywords:** aerobic, non-motile, Gram-positive, non-sporeforming, non-haemolytic, heterotrophic, mesophilic, squalene-degrading

## Abstract

*Corynebacterium terpenotabidum* Takeuchi *et. al* 1999 is a member of the genus *Corynebacterium*, which contains Gram-positive and non-spore forming bacteria with a high G+C content. *C. terpenotabidum* was isolated from soil based on its ability to degrade squalene and belongs to the aerobic and non-hemolytic *Corynebacteria*. It displays tolerance to salts (up to 8%) and is related to *Corynebacterium variabile* involved in cheese ripening. As this is a type strain of *Corynebacterium*, this project describing the 2.75 Mbp long chromosome with its 2,369 protein-coding and 72 RNA genes will aid the ***G****enomic* ***E****ncyclopedia of* ***Bacteria**** and* ***Archaea***** project.

## Introduction

Strain Y-11^T^ (= DSM 444721^T^) is the type strain of the species *Corynebacterium terpenotabidum* [1]. It was originally isolated from soil, although the exact source has not been published [2,3]. The genus *Corynebacterium* is comprised of Gram-positive bacteria with a high G+C content. It currently contains over 80 members [4] isolated from diverse backgrounds like human clinical samples [5] and animals [6], but also from soil [7] and ripening cheese [8].

Within this diverse genus, *C. terpenotabidum* has been proposed to form a subclade together with *C. variabile* DSM 20132^T^ and *C. nuruki* S6-4^T^, demonstrating 97.4% and 95.9% similarity respectively between the 16S rRNA gene sequences. Information on the strain is scarce. It was isolated for its ability to metabolize the linear triterpene squalene and classified as an *Arthrobacter* species [2,3], but no further information on the strain was supplied. Neither the origin nor the exact isolation procedures were reported. *C. terpenotabidum* can cleave squalene yielding geranylacetone [2] but also accepts some squalene derivatives [3].

Here we present a summary classification and a set of features for *C. terpenotabidum* DSM 44721^T^, together with the description of the genomic sequencing and annotation.

## Classification and features

A representative genomic 16S rRNA sequence of *C. terpenotabidum* DSM 44721^T^ was compared to the Ribosomal Database Project database [9]. *C. terpenotabidum* shows highest similarity to *C. variabile* (97.4%).

**Figure 1:**
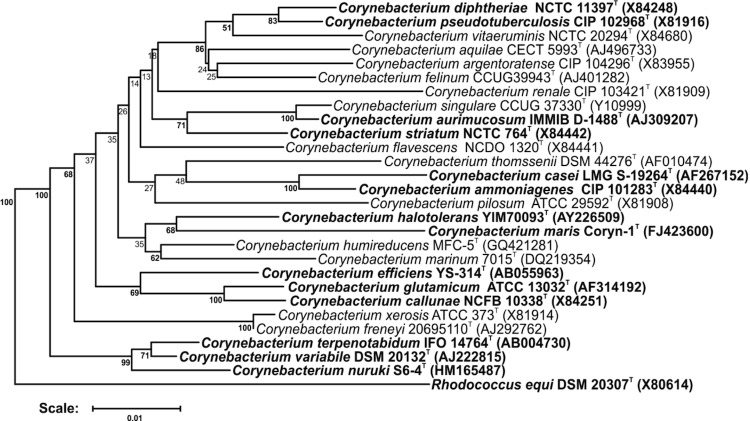
Phylogenetic tree highlighting the position of *C. terpenotabidum* relative to type strains of other species within the genus *Corynebacterium*. Species with at least one publicly available genome sequence (not necessarily the type strain) are highlighted in **bold face**. The tree is based on sequences aligned by the RDP aligner and utilizes the Jukes-Cantor corrected distance model to construct a distance matrix based on alignment model positions without alignment inserts, using a minimum comparable position of 200. The tree is built with RDP Tree Builder, which utilizes the Weighbor method [10] with an alphabet size of 4 and length size of 1,000. The building of the tree also involves a bootstrapping process repeated 100 times to generate a majority consensus tree [11]. *Rhodococcus equi* (X80614) was used as an outgroup.

Figure 1 shows the phylogenetic neighborhood of *C. terpenotabidum* in a 16S rRNA based tree. Within the genus *Corynebacterium, C. terpenotabidum* forms a distinct subclade together with *C. variabile* and *C. nuruki*.

**Figure 2:**
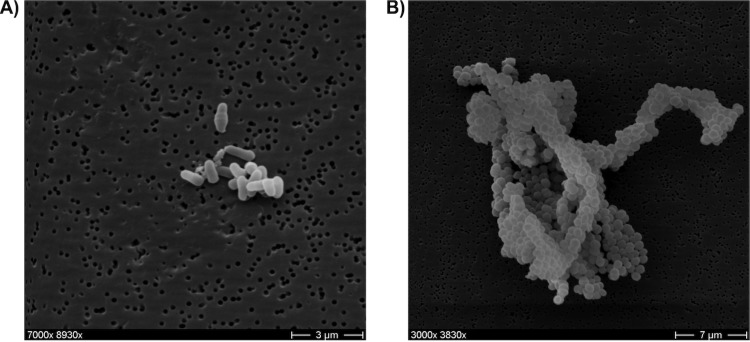
Scanning electron micrograph of *C. terpenotabidum* Y-11^T^. A) Free-floating cells. B) Aggregated cells.

*C. terpenotabidum* Y-11^T^ cells are Gram-positive non acid fast rods (1.0-1.5 μm x 0.5-0.8 μm wide) that grow strictly aerobically in rough, grayish-white colonies without diffusible pigments or aerial mycelia [1], [Table 1]. Cells grow with a wax-like quality on solid medium and tend to clot in liquid culture. Scanning electron micrograph pictures of liquid grown cultures revealed slight morphological differences between free-floating cells and clotted cells (Figure 2).

**Table 1:** Classification and general features of *C. terpenotabidum* Y-11^T^ according to the MIGS recommendations [12].

**MIGS ID**	**Property**	**Term**	**Evidence code^a)^**
	Current classification	Domain *Bacteria*	TAS [13]
		Phylum *Actinobacteria*	TAS [14]
		Class *Actinobacteria*	TAS [15]
		Order *Actinomycetales*	TAS [15-18]
		Family *Corynebacteriaceae*	TAS [15-17,19]
		Genus *Corynebacterium*	TAS [15-17,20,21]
		Species *Corynebacterium terpenotabidum*	TAS [1]
		Type-strain Y-11^T^ (=DSM 44721^T^)	TAS [1]
	Gram stain	positive	TAS [1]
	Cell shape	rod-shaped	TAS [1]
	Motility	non-motile	TAS [1]
	Sporulation	non-sporulating	TAS [1]
	Temperature range	mesophile	TAS [1]
	Optimum temperature	28°C	TAS [1]
	Salinity	0-8% (w/v) NaCl	TAS [1]
MIGS-22	Oxygen requirement	aerobe	TAS [1]
	Carbon source	fructose, galactose, mannose, lactate, ethanol	TAS [1]
	Energy metabolism	chemoorganoheterotrophic	NAS
	Terminal electron acceptor	oxygen	NAS
MIGS-6	Habitat	soil	TAS [2]
MIGS-15	Biotic relationship	free-living	NAS
MIGS-14	Pathogenicity	non-pathogenic	NAS
	Biosafety level	1	NAS
MIGS-23.1	Isolation	not reported	
MIGS-4	Geographic location	not reported	
MIGS-5	Sample collection time	not reported	
MIGS-4.1	Latitude	not reported	
MIGS-4.2	Longitude		
MIGS-4.3	Depth	not reported	
MIGS-4.4	Altitude	not reported	

a) Evidence codes - TAS: Traceable Author Statement (i.e., a direct report exists in the literature); NAS: Non-traceable Author Statement (i.e., not directly observed for the living, isolated sample, but based on a generally accepted property for the species, or anecdotal evidence). These evidence codes are from of the Gene Ontology project [22].

*C. terpenotabidum* was found to be able to utilize fructose, galactose, mannose, lactate, and ethanol as carbon source, while many others like arginine, aspartate, histidine, methylamine, ethylamine, methanol, galactose, lactose, maltose, sucrose, glycerol, sorbitol, mannitol, inositol, citrate, succinate, malonate, pimelate, *m-*hydroxybenzoate and *p*-hydroxybenzoate cannot be used. Optimal growth of strain Y-11^T^ is reported at 28°C. *C. terpenotabidum* was shown to grow with a salinity between 0 and 8.0% (w/v NaCl), with no growth at 10% [1]. The biochemical characterization revealed positive signals for urease, catalase, and hydrolysis of Tween 80.

### Chemotaxonomy

The cell wall of *C. terpenotabidum* Y-11^T^ contains alanine, glutamic acid, and meso-diaminopimelic acid in a molar ratio of 2.12: 1.00: 0.97. The main components of the cell wall sugars are described to be arabinose, galactose, and mannose in a molar ratio of 2.47: 1.71: 1.00. The glycan moiety of the cell wall was found to contain acetyl residues [1].

In *C. terpenotabidum*, cellular fatty acids are composed mainly of oleic acid (C_18:1_*ω9c*, 31%), palmitic acid (C_16:0_, 28%), and tuberculostearic acid 10-methyl (C_18:0_, 21%). The whole-cell methanolysate of strain Y-11 contained mycolic esters [1]. The predominant isoprenoid quinone is menaquinone MK-9(H_2_).

## Genome sequencing and annotation

### Genome project history

*C. terpenotabidum* Y-11^T^ was selected for sequencing as part of a project to define the core genome and pan genome of the non-pathogenic corynebacteria. While not being part of the ***G****enomic* ***E****ncyclopedia of* ***Bacteria**** and* ***Archaea***** (GEBA) project [23], sequencing of the type strain will nonetheless aid the GEBA effort. The genome project is deposited in the Genomes OnLine Database [24] and the complete genome sequence is deposited in GenBank. Sequencing, finishing and annotation were performed by the Center of Biotechnology (CeBiTec). A summary of the project information is shown in Table 2.

**Table 2:** Genome sequencing project information

**MIGS ID**	**Property**	**Term**
MIGS-31	Finishing quality	Finished
MIGS-28	Libraries used	Two genomic libraries: one 454 pyrosequencing PE library (3.4 kb insert sizes), one Illumina library
MIGS-29	Sequencing platforms	454 GS FLX Titanium, Illumina MiSeq
MIGS-31.2	Sequencing coverage	29.52× Pyrosequencing; 61.71 × SBS
MIGS-30	Assemblers	Newbler version 2.3
MIGS-32	Gene calling method	GeneMark, Glimmer
	INSDC ID	CP003696
	GenBank Date of Release	September 1, 2013 / after publication
	GOLD ID	Gi18852
	NCBI project ID	168617
MIGS-13	Source material identifier	DSM 44721
	Project relevance	Industrial, GEBA

### Growth conditions and DNA isolation

*C. terpenotabidum* strain Y-11^T^, DSM 44721, was grown aerobically in LB broth (Carl Roth GmbH, Karlsruhe,Germany) at 30 °C. DNA was isolated from ~ 10^8^ cells using the protocol described by Tauch *et al*. 1995 [25].

### Genome sequencing and assembly

The genome was sequenced using a 454 sequencing platform. A standard 3k paired end sequencing library was prepared according to the manufacturers protocol (Roche). The genome was sequenced using the GS-FLX platform with Titanium chemistry, yielding 384,252 total reads, providing 29.52× coverage of the genome. Pyrosequencing reads were assembled using the Newbler assembler v2.3 (Roche). The initial Newbler assembly consisted of 22 contigs in six scaffolds. Analysis of the six scaffolds revealed five that made up the chromosome, while the remaining one contained five copies of the RRN operon that caused the scaffold breaks. The scaffolds were ordered based on alignments to the complete genomes of *C. variabile* [26] and subsequent verification by restriction digestion, Southern blotting and hybridization with a 16S rDNA specific probe.

The Phred/Phrap/Consed software package [27-30] was used for sequence assembly and quality assessment in the subsequent finishing process. After the shotgun stage, gaps between contigs were closed by editing in Consed (for repetitive elements) and by PCR with subsequent Sanger sequencing (IIT Biotech GmbH, Bielefeld, Germany). A total of 12 additional reactions were necessary to close gaps not caused by repetitive elements.

To raise the quality of the assembled sequence, Illumina reads were used to correct potential base errors and increase consensus quality. A WGS library was prepared using the Illumina-Compatible Nextera DNA Sample Prep Kit (Epicentre, WI, U.S.A) according to the manufacturer's protocol. The library was sequenced in a 2x 120 bp paired read run on the MiSeq platform, yielding 2,307,926 total reads. Together, the combination of the Illumina and 454 sequencing platforms provided 91.2× coverage of the genome.

### Genome annotation

Gene prediction and annotation were done using the PGAAP pipeline [31]. Genes were identified using GeneMark [32], GLIMMER [33], and Prodigal [34]. For annotation, BLAST searches against the NCBI Protein Clusters Database [35] are performed and the annotation is enriched by searches against the Conserved Domain Database [36] and subsequent assignment of coding sequences to COGs. Non-coding genes and miscellaneous features were predicted using tRNAscan-SE [37], Infernal [38], RNAMMer [39], Rfam [40], TMHMM [41], and SignalP [42].

## Genome properties

**Figure 3:**
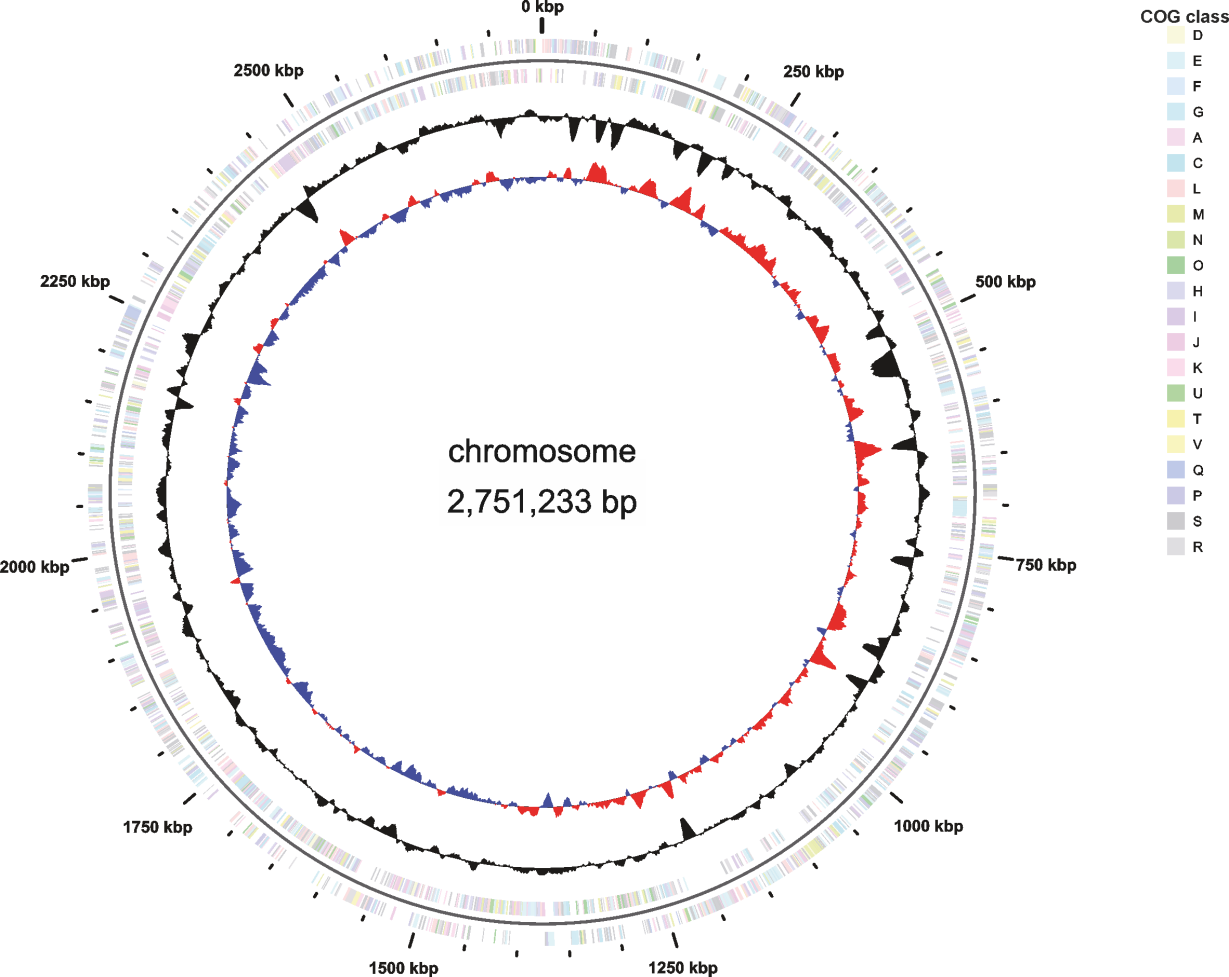
Graphical map of the chromosome. From the outside in: Genes on forward strand (colored according to COG categories), Genes on reverse strand (colored according to COG categories), GC content, GC skew.

The genome consists of one circular chromosome of 2,751,233 bp (67.02% G+C content) with no additional extrachromosomal elements present. A total of 2,441 genes were predicted, 2,369 of which are protein coding genes. 1,306 (55.13%) of the protein coding genes were assigned to a putative function with the remaining annotated as hypothetical proteins. In addition, 910 protein coding genes belong to 281 paralogous families in this genome, corresponding to a gene content redundancy of 38.41% [Figure 3]. The properties and the statistics of the genome are summarized in Table 3, and Table 4.

**Table 3:** Genome Statistics

**Attribute**	Value	% of total^a^
Genome size (bp)	2,751,233	100.00
DNA coding region (bp)	2,441,394	88.74
DNA G+C content (bp)	1,843,810	67.02
Total genes	2,441	100.00
RNA genes	72	2.96
rRNA operons	5	
tRNA genes	57	2.34
Protein-coding genes	2,369	97.04
Genes with function prediction (protein)	1,306	55.13
Genes assigned to COGs	1,812	74.23
Genes in paralog clusters	910	38.41
Genes with signal peptides	224	9.54
Genes with transmembrane helices	606	25.58

a) The total is based on either the size of the genome in base pairs or the total number of genes in the annotated genome.

**Table 4:** Number of genes associated with the general COG functional categories

**Code**	**Value**	**%age**	**Description**
J	151	6.37	Translation, ribosomal structure and biogenesis
A	1	0.04	RNA processing and modification
K	152	6.42	Transcription
L	136	5.74	Replication, recombination and repair
B	0	0.00	Chromatin structure and dynamics
D	20	0.84	Cell cycle control, cell division, chromosome partitioning
Y	0	0.00	Nuclear structure
V	32	1.35	Defense mechanisms
T	58	2.45	Signal transduction mechanisms
M	81	3.42	Cell wall/membrane biogenesis
N	1	0.04	Cell motility
Z	0	0.00	Cytoskeleton
W	0	0.00	Extracellular structures
U	26	1.10	Intracellular trafficking and secretion, and vesicular transport
O	72	3.04	Posttranslational modification, protein turnover, chaperones
C	127	5.36	Energy production and conversion
G	115	4.85	Carbohydrate transport and metabolism
E	218	9.20	Amino acid transport and metabolism
F	68	2.87	Nucleotide transport and metabolism
H	97	4.09	Coenzyme transport and metabolism
I	121	5.11	Lipid transport and metabolism
P	151	6.37	Inorganic ion transport and metabolism
Q	76	3.21	Secondary metabolites biosynthesis, transport and catabolism
R	274	11.57	General function prediction only
S	138	5.83	Function unknown
-	557	23.51	Not in COGs

## References

[1] Takeuchi M Sakane T Nihira T Yamada Y Imai K *Corynebacterium terpenotabidum* sp. nov., a bacterium capable of degrading squalene. Int J Syst Bacteriol 1999; 49:223-229 10.1099/00207713-49-1-223 10028267

[2] Yamada Y Motoi H Kinoshita S Takada N Okada H Oxidative degradation of squalene by *Arthrobacter* species. Appl Microbiol 1975; 29:400-404 1115507 10.1128/am.29.3.400-404.1975 PMC186987

[3] Yamada Y Kusuhara N Okada H Oxidation of linear terpenes and squalene variants by *Arthrobacter sp.* Appl Environ Microbiol 1977; 33:771-776 869527 10.1128/aem.33.4.771-776.1977 PMC170765

[4] Euzéby JP List of Bacterial Names with Standing in Nomenclature: a folder available on the Internet. Int J Syst Bacteriol 1997; 47:590-592 10.1099/00207713-47-2-590 9103655

[5] Renaud FNR Aubel D Riegel P Meugnier H Bollet C *Corynebacterium freneyi* sp. nov., alpha-glucosidase-positive strains related to *Corynebacterium xerosis*. Int J Syst Evol Microbiol 2001; 51:1723-1728 10.1099/00207713-51-5-1723 11594602

[6] Collins MD Hoyles L Foster G Falsen E *Corynebacterium caspium* sp. nov., from a Caspian seal (Phoca caspica). Int J Syst Evol Microbiol 2004; 54:925-928 10.1099/ijs.0.02950-0 15143043

[7] Zhou Z Yuan M Tang R Chen M Lin M Zhang W *Corynebacterium deserti* sp. nov., isolated from desert sand. Int J Syst Evol Microbiol 2012; 62:791-794 10.1099/ijs.0.030429-0 21571935

[8] Brennan NM Brown R Goodfellow M Ward AC Beresford TP Simpson PJ Fox PF Cogan TM *Corynebacterium mooreparkense* sp. nov. and *Corynebacterium casei* sp. nov., isolated from the surface of a smear-ripened cheese. Int J Syst Evol Microbiol 2001; 51:843-852 10.1099/00207713-51-3-843 11411705

[9] Cole JR Wang Q Cardenas E Fish J Chai B Farris RJ Kulam-Syed-Mohideen AS McGarrell DM Marsh T Garrity GM The Ribosomal Database Project: improved alignments and new tools for rRNA analysis. Nucleic Acids Res 2009; 37(Database issue):D141-D145 10.1093/nar/gkn879 19004872 PMC2686447

[10] Bruno WJ Socci ND Halpern AL Weighted neighbor joining: a likelihood-based approach to distance-based phylogeny reconstruction. Mol Biol Evol 2000; 17:189-197 10.1093/oxfordjournals.molbev.a026231 10666718

[11] Cole JR Chai B Farris RJ Wang Q Kulam-Syed-Mohideen AS McGarrell DM Bandela AM Cardenas E Garrity GM Tiedje JM The ribosomal database project (RDP-II): introducing myRDP space and quality controlled public data. Nucleic Acids Res 2007; 35(Database issue):D169-D172 10.1093/nar/gkl889 17090583 PMC1669760

[12] Field D Garrity G Gray T Morrison N Selengut J Sterk P Tatusova T Thomson N Allen MJ Angiuoli SV The minimum information about a genome sequence (MIGS) specification. Nat Biotechnol 2008; 26:541-547 10.1038/nbt1360 18464787 PMC2409278

[13] Woese CR Kandler O Wheelis ML Towards a natural system of organisms: proposal for the domains Archaea, Bacteria, and Eucarya. Proc Natl Acad Sci USA 1990; 87:4576-4579 10.1073/pnas.87.12.4576 2112744 PMC54159

[14] Garrity GM, Holt JG. The Road Map to the Manual. In: Garrity GM, Boone DR, Castenholz RW (eds), Bergey's Manual of Systematic Bacteriology, Second Edition, Volume 1, Springer, New York, 2001, p. 119-169.

[15] Stackebrandt E Rainey FA Ward-Rainey NL Proposal for a New Hierarchic Classification System, *Actinobacteria* classis nov. Int J Syst Bacteriol 1997; 47:479-491 10.1099/00207713-47-2-479

[16] Zhi XY Li WJ Stackebrandt E An update of the structure and 16S rRNA gene sequence-based definition of higher ranks of the class Actinobacteria, with the proposal of two new suborders and four new families and emended descriptions of the existing higher taxa. Int J Syst Evol Microbiol 2009; 59:589-608 10.1099/ijs.0.65780-0 19244447

[17] Skerman VBD McGowan V Sneath PHA Approved Lists of Bacterial Names. Int J Syst Bacteriol 1980; 30:225-420 10.1099/00207713-30-1-225 20806452

[18] Buchanan RE Studies in the nomenclature and classification of bacteria. II. The primary subdivisions of the Schizomycetes. J Bacteriol 1917; 2:155-164 16558735 10.1128/jb.2.2.155-164.1917 PMC378699

[19] Lehmann KB, Neumann R. Lehmann's Medizin, Handatlanten. X Atlas und Grundriss der Bakteriologie und Lehrbuch der speziellen bakteriologischen Diagnostik., Fourth Edition, Volume 2, J.F. Lehmann, München, 1907, p. 270.

[20] Lehmann KB, Neumann R. Atlas und Grundriss der Bakteriologie und Lehrbuch der speziellen bakteriologischen Diagnostik, First Edition, J.F. Lehmann, München, 1896, p. 1-448.

[21] Bernard KA Wiebe D Burdz T Reimer A Ng B Singh C Schindle S Pacheco AL Assignment of *Brevibacterium stationis* (ZoBell and Upham 1944) Breed 1953 to the genus *Corynebacterium*, as *Corynebacterium stationis*** comb. nov., and emended description of the genus *Corynebacterium* to include isolates that can alkalinize citrate. Int J Syst Evol Microbiol 2010; 60:874-879 10.1099/ijs.0.012641-0 19661509

[22] Ashburner M Ball CA Blake JA Botstein D Butler H Cherry JM Davis AP Dolinski K Dwight SS Eppig JT Gene ontology: tool for the unification of biology. The Gene Ontology Consortium. Nat Genet 2000; 25:25-29 10.1038/75556 10802651 PMC3037419

[23] Wu D Hugenholtz P Mavromatis K Pukall R Dalin E Ivanova NN Kunin V Goodwin L Wu M Tindall BJ A phylogeny-driven genomic encyclopaedia of *Bacteria* and *Archaea.* Nature 2009; 462:1056-1060 10.1038/nature08656 20033048 PMC3073058

[24] Liolios K Chen IM Mavromatis K Tavernarakis N Hugenholtz P Markowitz VM Kyrpides NC The Genomes OnLine Database (GOLD) in 2009: status of genomic and metagenomic projects and their associated metadata. Nucleic Acids Res 2010; 38:D346-D354 10.1093/nar/gkp848 19914934 PMC2808860

[25] Tauch A Kassing F Kalinowski J Pühler A The *Corynebacterium xerosis* composite transposon Tn5432 consists of two identical insertion sequences, designated IS1249, flanking the erythromycin resistance gene *ermCX*. Plasmid 1995; 34:119-131 10.1006/plas.1995.9995 8559800

[26] Schröder J Maus I Trost E Tauch A Complete genome sequence of *Corynebacterium variabile* DSM 44702 isolated from the surface of smear-ripened cheeses and insights into cheese ripening and flavor generation. BMC Genomics 2011; 12:545 10.1186/1471-2164-12-545 22053731 PMC3219685

[27] Ewing B Green P Base-calling of automated sequencer traces using phred. II. Error probabilities. Genome Res 1998; 8:175-185 10.1101/gr.8.3.175 9521922

[28] Gordon D Abajian C Green P Consed: a graphical tool for sequence finishing. Genome Res 1998; 8:195-202 10.1101/gr.8.3.195 9521923

[29] Gordon D. Viewing and editing assembled sequences using Consed. Curr Protoc Bioinformatics 2003;Chapter 11:Unit11 2. 10.1002/0471250953.bi1102s02 18428695

[30] Ewing B Hillier L Wendl MC Green P Base-calling of automated sequencer traces using phred. I. Accuracy assessment. Genome Res 1998; 8:175-185 10.1101/gr.8.3.175 9521921

[31] NCBI. 2010 NCBI Prokaryotic Genomes Automatic Annotation Pipeline (PGAAP). <http://www.ncbi.nlm.nih.gov/genomes/static/Pipeline.html>.

[32] Borodovsky M, Mills R, Besemer J, Lomsadze A. Prokaryotic gene prediction using GeneMark and GeneMark.hmm. Curr Protoc Bioinformatics 2003;Chapter 4:Unit4 5. 10.1002/0471250953.bi0405s01 18428700

[33] Delcher AL Harmon D Kasif S White O Salzberg SL Improved microbial gene identification with GLIMMER. Nucleic Acids Res 1999; 27:4636-4641 10.1093/nar/27.23.4636 10556321 PMC148753

[34] Hyatt D Chen GL Locascio PF Land ML Larimer FW Hauser LJ Prodigal: prokaryotic gene recognition and translation initiation site identification. BMC Bioinformatics 2010; 11:119 10.1186/1471-2105-11-119 20211023 PMC2848648

[35] Klimke W Agarwala R Badretdin A Chetvernin S Ciufo S Fedorov B Kiryutin B O'Neill K Resch W Resenchuk S The National Center for Biotechnology Information's Protein Clusters Database. Nucleic Acids Res 2009; 37(Database issue):D216-D223 10.1093/nar/gkn734 18940865 PMC2686591

[36] Marchler-Bauer A Anderson JB Chitsaz F Derbyshire MK DeWeese-Scott C Fong JH Geer LY Geer RC Gonzales NR Gwadz M CDD: specific functional annotation with the Conserved Domain Database. Nucleic Acids Res 2009; 37(Database issue):D205-D210 10.1093/nar/gkn845 18984618 PMC2686570

[37] Lowe TM Eddy SR tRNAscan-SE: a program for improved detection of transfer RNA genes in genomic sequence. Nucleic Acids Res 1997; 25:955-964 9023104 10.1093/nar/25.5.955 PMC146525

[38] Eddy SR A memory-efficient dynamic programming algorithm for optimal alignment of a sequence to an RNA secondary structure. BMC Bioinformatics 2002; 3:18 10.1186/1471-2105-3-18 12095421 PMC119854

[39] Lagesen K Hallin P Rodland EA Staerfeldt HH Rognes T Ussery DW RNAmmer: consistent and rapid annotation of ribosomal RNA genes. Nucleic Acids Res 2007; 35:3100-3108 10.1093/nar/gkm160 17452365 PMC1888812

[40] Griffiths-Jones S, Moxon S, Marshall M, Khanna A, Eddy SR, Bateman A. Rfam: annotating non-coding RNAs in complete genomes. *Nucleic Acids Res* 2005;**33** (Database Issue):D121-124. 10.1093/nar/gki081 PMC540035 15608160

[41] Krogh A Larsson B von Heijne G Sonnhammer EL Predicting transmembrane protein topology with a hidden Markov model: application to complete genomes. J Mol Biol 2001; 305:567-580 10.1006/jmbi.2000.4315 11152613

[42] Bendtsen JD Nielsen H von Heijne G Brunak S Improved prediction of signal peptides: SignalP 3.0. J Mol Biol 2004; 340:783-795 10.1016/j.jmb.2004.05.028 15223320

